# Identification of *Aspergillus terreus* and *Aspergillus pseudonomiae* as causative agents of aspergillosis in endangered Okinawa Rails

**DOI:** 10.3389/fvets.2025.1675145

**Published:** 2025-12-22

**Authors:** Sofia Marisel Rivelli Zea, Yumiko Nakaya, Hiroki Takahashi, Takashi Nagamine, Takashi Yaguchi, Takahito Toyotome

**Affiliations:** 1 Department of Veterinary Medicine, Obihiro University of Agriculture and Veterinary Medicine, Obihiro, Hokkaido, Japan; 2 Laboratory of Veterinary Diagnostic and Veterinary Products Control, National Service for Quality and Animal Health (SENACSA), San Lorenzo, Paraguay; 3 Okinawa Wildlife Federation, Okinawa, Japan; 4 Medical Mycology Research Center, Chiba University, Chiba, Japan; 5 Diagnostic Center of Animal Health and Food Safety, Obihiro University of Agriculture and Veterinary Medicine, Obihiro, Hokkaido, Japan; 6 Department of Pharmaceutical Sciences, School of Pharmacy at Narita, International University of Health and Welfare, Narita, Japan

**Keywords:** Okinawa Rail, *Aspergillus terreus*, *Aspergillus pseudonomiae*, aspergillosis, aflatoxin, endangered birds

## Abstract

**Introduction:**

Aspergillosis is a serious infectious disease in avian species, including endangered birds. However, reports in such species remain scarce. This study focused on the Okinawa Rail (*Hypotaenidia okinawae*), an endangered flightless bird restricted to the northern forests of Okinawa Island, Japan.

**Methods:**

Air sac tissues and swabs from two deceased Okinawa Rails were cultured on potato dextrose agar. Identification of isolates was confirmed by ITS and *benA* sequencing. Antifungal susceptibility was tested using CLSI M38 broth microdilution. Aflatoxin production of isolates was assessed by dichlorvos–ammonia method and quantified via ELISA and LC–MS. Genomic DNA from these isolates was extracted and sequenced using PacBio technology, followed by genome assembly and analysis of antifungal resistance genes and secondary metabolite clusters.

**Results and Discussion:**

We identified and characterized *Aspergillus terreus* and *Aspergillus pseudonomiae* as causative agents of aspergillosis in two captive Okinawa Rails. In Case 1, an *A*. *terreus* isolate showed low susceptibility to voriconazole. It remains unclear whether this low susceptibility was intrinsic or acquired during antifungal treatment. Two phenotypically distinct isolates of *A*. *pseudonomiae* were obtained from the second case. One of them lacked sclerotia and aflatoxin production, suggesting a possible adaptation during infection. This is the first report of *A*. *pseudonomiae* isolation from animal hosts in Japan. Accurate identification and genomic analysis of *Aspergillus* isolates provide insights into antifungal resistance and the ecological dynamics of aspergillosis in Okinawa’s forest environment, with important implications for wildlife conservation.

## Introduction

The Okinawa Rail (*Hypotaenidia okinawae*), which was discovered by the Yamashina Institute for Ornithology in 1981, is a species restricted to the northern forest areas of Okinawa Island and is the only flightless bird in Japan (1). The Okinawa Rail plays a crucial role in the ecosystem of the northern forest areas of Okinawa Island. As a primary consumer, it contributes to the control of certain insect populations and the dispersal of seeds, thereby influencing the structure and diversity of the forest (2). The species has been listed as Endangered in the IUCN Red List since 1994 due to its very small population in a specific part of Okinawa Island (3). When the species was discovered, around 1,800 birds were thought to exist, but due to factors such as road accidents, changes in the forest environment, and predation by introduced species such as mongoose and feral cats, the population decreased. By 2005, approximately 700 individuals remained, placing the species one among those at the highest risk of extinction in Japan (4, 5). Accordingly, cooperative efforts based on the Ministry of the Environment’s conservation and breeding program are being undertaken by the government, NGOs, researchers, and local residents to promote conservation measures both within and outside the habitat, which has led to an encouraging recovery of the Okinawa Rail, with the population now reaching around 1,500 individuals (6).

Aspergillosis, which is caused by *Aspergillus* species, is one of the major fungal infectious diseases in avian species. Although *Aspergillus fumigatus* is the most prevalent etiologic agent of avian aspergillosis, *A. flavus* and *A. terreus* are also recognized as notable causative agents of the disease (7–10). *A. flavus* has been isolated in aspergillosis cases of various avian species involving from broiler chicks (11), falcons (12, 13), a parrot (14), and a royal tern *Thalasseus maximus* (15). *A. terreus* was also isolated from falcons (12, 16), a caged pigeon (17), and free-living goshawks (18). Aspergillosis can induce severe pathological effects on birds, including respiratory distress, weight loss, and, in advanced cases, can lead to mortality. The disease is typically contracted through the inhalation of fungal spores, which are ubiquitous in the environment, especially in the soil (19, 20). This puts ground-dwelling birds such as the Okinawa Rail at risk. However, cases of aspergillosis in wild Okinawa Rails have not been reported. Compared to populations in the wild, birds in captivity are more predisposed to the development of aspergillosis due to the stress of confined conditions, the use of antibiotics in the treatment of other diseases, the stress of the treatment itself, and the rearing environment that differs from that in the wild. Recently, a case of aspergillosis caused by *A. flavus* in a captive Okinawa Rail was reported by Kano et al. (21). Generally, the diagnosis of aspergillosis in birds involves the evaluation of clinical signs, imaging, and laboratory tests for veterinary treatment, which often includes antifungal medication (22, 23). Prevention is the most effective strategy, which requires maintaining clean and stress-free environments for captive birds, including Okinawa Rails (24). Further research is needed to fully understand the susceptibility of Okinawa Rails to aspergillosis and develop effective conservation strategies. This study combines clinical observations with laboratory and genomic investigations to provide comprehensive insights into aspergillosis in Okinawa Rails. Here, two cases of aspergillosis in captive Okinawa Rails are discussed, the causative agents are characterized in detail, and scaffold-level assembled genomes are obtained.

## Materials and methods

### Ethical considerations regarding the use of animals

Health checks and all diagnostic and treatment procedures were conducted in accordance with the practice guidelines of the Okinawa Wildlife Federation. Experiments using Okinawa Rail tissue samples obtained at necropsy were performed under the regulations of the Management and Operation of Animal Experiments by the Animal Care and Use Committee of the Obihiro University of Agriculture and Veterinary Medicine.

### Isolation and molecular identification of causative agents

Air sac tissues and cotton swab samples rubbed with infection foci inside and outside the air sac were cultured on potato dextrose agar (PDA, Becton, Dickinson and Co., Franklin Lakes, NJ, United States) at 35 °C for 2 days. The colonies were streaked onto another agar medium and used for further experiments. Molecular identification of the isolated *Aspergillus* species was performed as described (1). In the present study, we analyzed ITS 1 and 2 and partial sequences of *benA*. The obtained sequences were analyzed using BLASTN (25).

### Antifungal susceptibility test

Minimum inhibitory concentrations (MICs) of antifungals against the isolates were determined using the broth microdilution method based on CLSI M38 3rd Edition (26), with slight modification by employing Dry Plate Eiken (Eiken Chemical Co., Ltd., Tokyo, Japan) as shown previously (27).

### Examination of aflatoxigenicity of isolates

Aflatoxigenicity was examined using the DV-AM method (28). The *A. oryzae* RIB40 and *A. flavus* NRRL3357 strains used as reference strains for aflatoxin detection in the experiment were provided by the Medical Mycology Research Center, Chiba University, with support in part by a procedure briefly described hereafter. The GY2-0.5 agar medium (2% (w/v) yeast extract, 0.5% (w/v) glucose, and 2% agar) was used for all experiments (29). Dichlorvos solution in methanol (4 mg/mL; AccuStandard, Inc., New Haven, CT, United States) was streaked on GY2-0.5 agar and then inoculated with each strain. After culturing at 25 °C and 35 °C for 3 days, aflatoxigenicity was detected by exposing fungal colonies to ammonia, which induces color development in aflatoxigenic strains. Quantification of total aflatoxins, including aflatoxins B1, G1, B2, and G2, was performed using the MycoJudge Total Aflatoxin Kit (NH Foods Ltd. R&D Center, Ibaraki, Japan). Spore suspensions were inoculated in 20 mL GY2-0.5 broth and incubated at 25 °C or 35 °C for 3 days. The filtrates of the culture supernatants were used for quantifying total aflatoxin.

### Determination of production and quantity of each aflatoxin compound from a. pseudonomiae strains

The filtrates of the culture supernatants used for quantifying total aflatoxin were analyzed by liquid chromatography–mass spectrometry (LC–MS), which was performed by NDTS Corp. (Hokkaido, Japan) as described below. First, 90 μL of the samples and standards were mixed with 160 μL of 0.5 M perchloric acid. For the quantification of aflatoxin B1, B2, G1, and G2, 10 μL of 10 ng/mL aflatoxin M1 solution was added with perchloric acid to the samples and standards as an internal standard. After mixing for 30 s, the mixture was centrifuged at 13,000 rpm for 5 min at 4 °C. The supernatant was collected and used for MS analysis. Mobile phase A comprised 10 mM ammonium acetate in a 5:95 mixture of methanol and water. Mobile phase B consisted of methanol. The flow rate was set to 0.2 mL/min. The gradient program for mobile phase B was as follows: starting at 0% and linearly increasing to 100% over 14.0 min, maintaining at 100% until 16.5 min, then returning to 0% by 17.0 min, and holding at 0% until 20.0 min. A Triart C18 column (150 mm × 2.1 mm i.d; YMC Co. Ltd., Kyoto, Japan) was used. The column temperature was maintained at 40 °C, and the injection volume was 10 μL. LCMS-8045 (Shimadzu Corporation, Kyoto, Japan) was used for MS analysis. The ionization mode was set to ESI (negative). The interface voltage was 4.0 kV. The desolvation line temperature was maintained at 250 °C. The heating gas flow rate was 10 L/min at 400 °C. The nebulizer gas flow rate was 180 L/h, and the drying gas flow rate was 10 L/min. The monitored ions and their respective transitions with collision energies are listed in Supplementary Table S1.

### Genomic DNA preparation, sequencing, and computational analyses

Genomic DNA was extracted from the isolates using a Genomic-tip 20/G (QIAGEN N. V., Venlo, The Netherlands). Briefly, spore suspensions were inoculated in 10 mL potato dextrose broth with 0.1% yeast extract and cultured at 35 °C for 24 h. Hyphal cells were recovered using Miracloth (Merck Millipore, Burlington, MT, United States), frozen, and crushed using a mortar and pestle. Cells were suspended in lysis buffer containing 100 U/mL ZymoLyase (Nacalai Tesque, Kyoto, Japan). Subsequent procedures were performed in accordance with the manufacturer’s instructions. Short-DNA elimination was performed using a short-read elimination kit (Pacific Biosciences, Menlo Park, CA, United States).

Purified genomic DNA from the isolates was processed by the Bioengineering Laboratory. Co., Ltd. (Kanagawa, Japan). Briefly, libraries were prepared using the SMRTbell Prep Kit 3.0 and SMRTbell gDNA Sample Amplification Kit (Pacific Biosciences) and sequenced using the Revio Polymerase Kit and Revio system (Pacific Biosciences). To align the sub-reads, overhang adaptor sequences were removed from the sequences obtained using SMRT Link (ver. 13.0.0.207600). From the sub-read data, each consensus was called. The consensus sequences with ≥ 20 quality values were used as HiFi reads in further analysis. Ultralow PCR adaptors were removed using Lima (ver. 2.9.0) and duplicate PCR reads were removed using pbmarkdup (ver. 1.0.3) from the Hi-Fi reads. Reads less than 1,000 bp in length were removed using FiltLong (ver. 0.2.1). The resulting reads were assembled using IPA software (ver. 1.8.0), and the completeness of the genome assembly was evaluated using BUSCO (ver. 5.4.1) (30) with the database Eurotiales _odb10. Gene prediction and annotation were performed using Funannotate software (Galaxy Version 1.8.15) (31) on the Galaxy Australia platform (32).

In accordance with the study by Nargesi et al. (33), the reference amino acid sequences of Cyp51A (gene ID: 4321797; ATEG_05917), Cyp51B (gene ID: 4317693; ATEG_02850), Cdr4 (gene ID: 4321786; ATEG_05640), Cdr4B (gene ID: 4319285; ATEG_07138), AtrR (gene ID: 4318923; ATEG_06908), FfmA (gene ID: 43159, ATEG_01517), Hmg1(gene ID: 4354193; ATEG_09520), and HapE (gene ID: 4321255; ATEG_05632) retrieved from NCBI and FungiDB were used to compare the corresponding regions of the *A. terreus* TT0037 strain. In addition, the reference amino acid sequence of the Cdr1B homolog (ATEG_00635) in *A. terreus* was compared with the corresponding sequence in *A. terreus* TT0037.

Chromeister (Galaxy Version 1.5.a) (34) on Galaxy Australia was used for pairwise genome comparison between the two *Aspergillus pseudonomiae* isolates from Case 2 and for visualization. DiGAlign was used to compare each homologous pair of scaffolds from *A. pseudonomiae* NS-type- and S-type strains (35). AntiSMASH (Galaxy Version 6.1.1) on Galaxy Australia was used to determine secondary metabolite biosynthetic gene clusters.

## Results

### Two aspergillosis cases in Okinawa Rails

A concise summary of the two cases is presented below; full clinical histories and treatment details are provided in the Supplementary Text.

### Case 1

**Figure 1:**
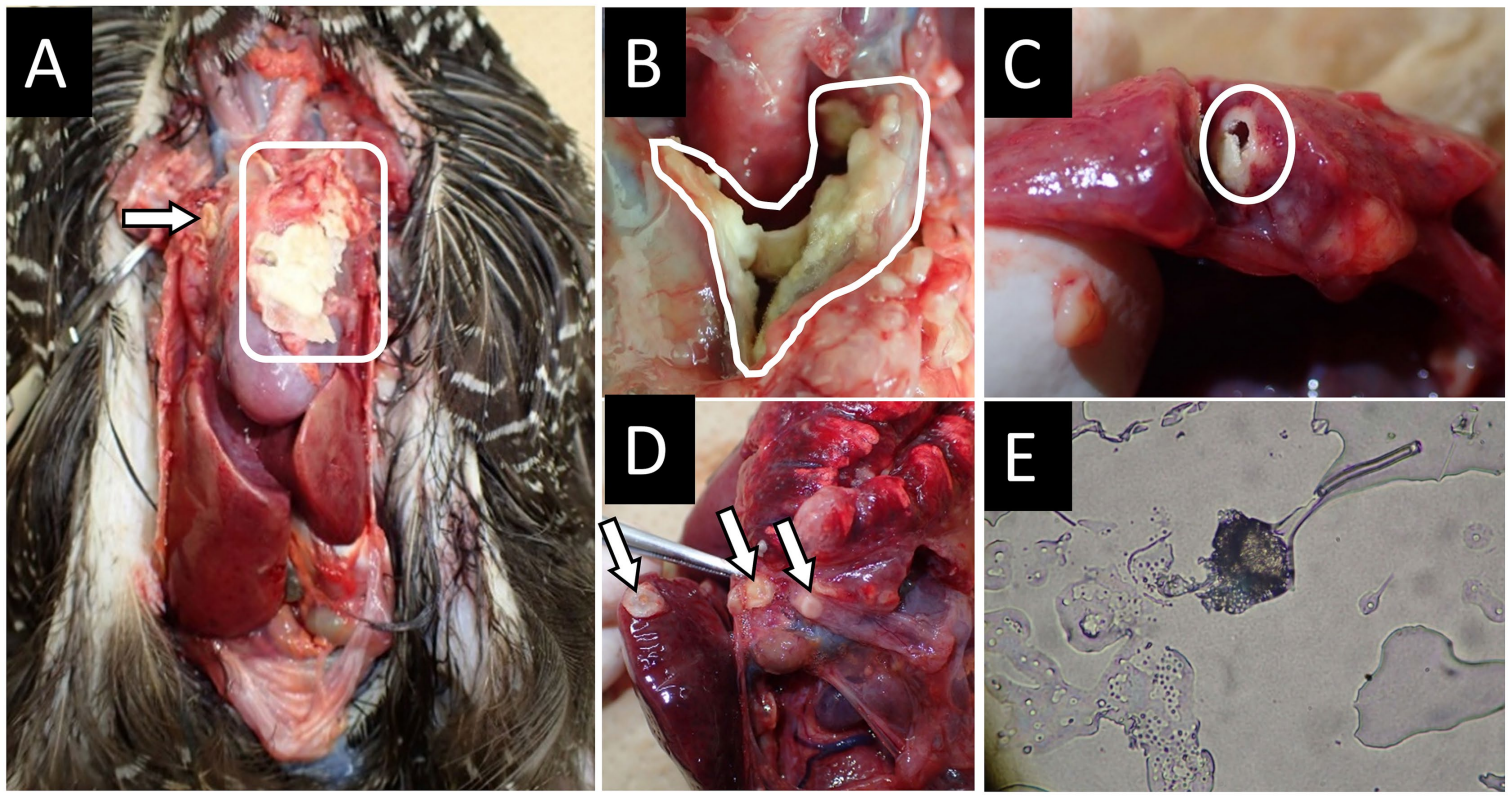
Necropsy findings in case 1 of an Okinawa Rail affected with aspergillosis. **(A)** Necropsy image of the Okinawa Rail, highlighting the thoracic cavity. The circled area indicates the presence of a large fungal infection focus. **(B)** Fungal mass found in the upper part of the heart. **(C)** Fungal mass found in the lung. **(D)** Another image focused on the thoracic cavity of the Okinawa Rail. Fungal masses were formed on multiple organs. The circled area and arrows in panels **A–D** indicate major fungal infection sites. **(E)** Microscopic examination of a sample taken from the fungal mass indicated in the circle of panel **A**. An aspergillum-like structure was observed in the sample.

A 13-year-old male Okinawa Rail (SB34) initially presented with respiratory symptoms and pneumonia, which improved after antibacterial therapy. From January 2020, progressive weight loss and respiratory distress observed. Despite sequential and overlapping antifungal treatments (including itraconazole, voriconazole, micafungin, and amphotericin B) and supportive care with oxygen therapy, the bird’s condition continue to deteriorate, ultimately resulting in death on June 7, 2020. Necropsy revealed extensive fungal colonization in air sacs, lungs, and multiple organs (circles and arrows in Figure 1).

### Case 2

**Figure 2:**
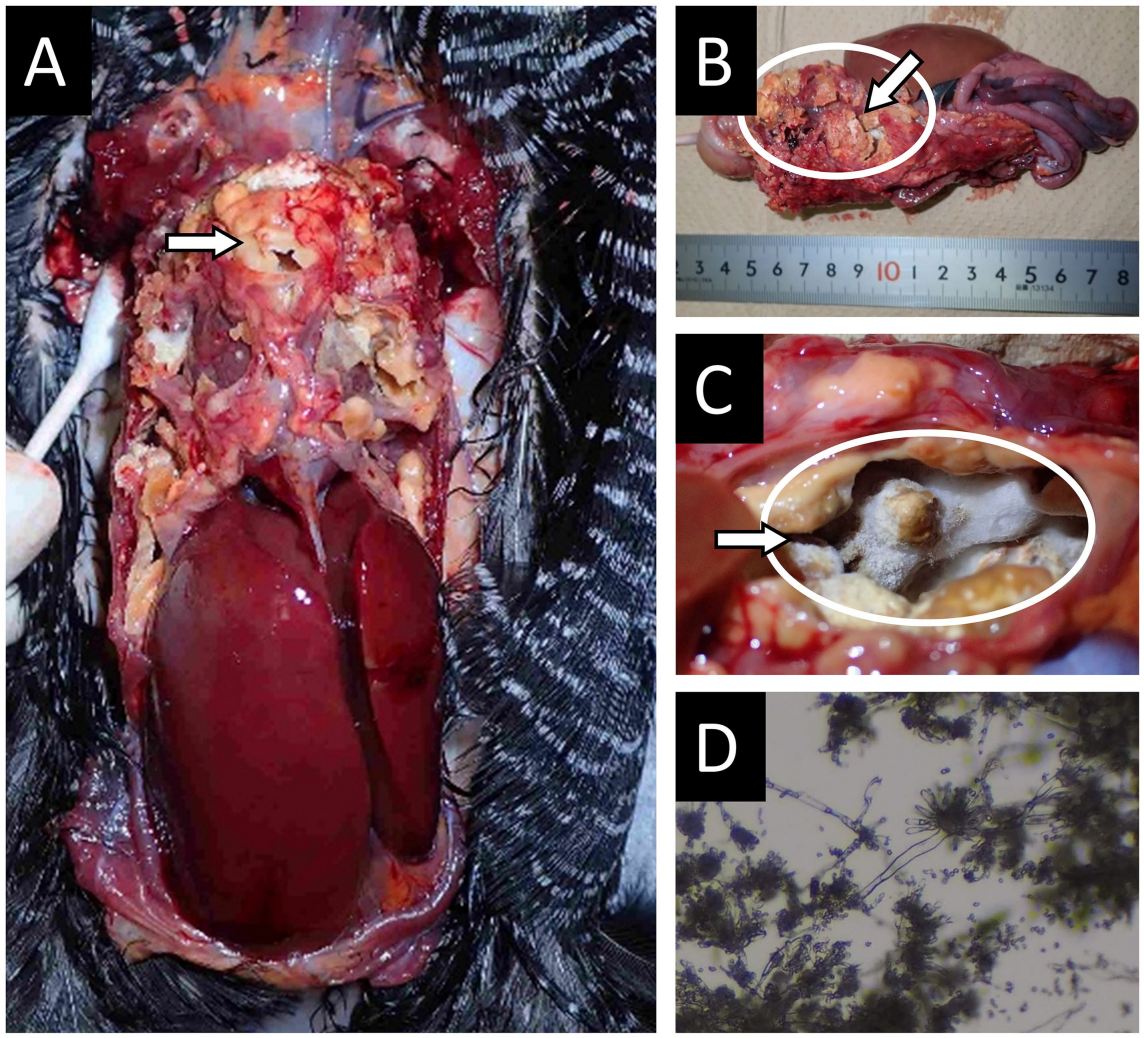
Necropsy findings in case 2 of an Okinawa Rail affected with aspergillosis. **(A)** Thoracic cavity of the Okinawa Rail. **(B)** Thoracic and abdominal cavities were occupied with large fungal masses. **(C)** Fungal mat within the posterior air sac. The circled area and arrows in panels **A–C** indicate major fungal infection sites. **(D)** Microscopic examination of a sample taken from a fungal mass from inside the air sac. Aspergillum-like structures are noted in the image.

A captive female Okinawa Rail (SB146), aged approximately 2 years and 10 months, exhibited severe respiratory distress in January 2021. Imaging and blood tests suggested aspergillosis, and intensive antifungal therapy combined with antibacterial agents was initiated under high-oxygen conditions. Despite aggressive treatment, the bird died on day 34 of illness. Necropsy revealed massive fungal masses occupying thoracic and abdominal cavities, air sacs, and lungs (arrows and circles in Figure 2).

### Causative agents

**Figure 3:**
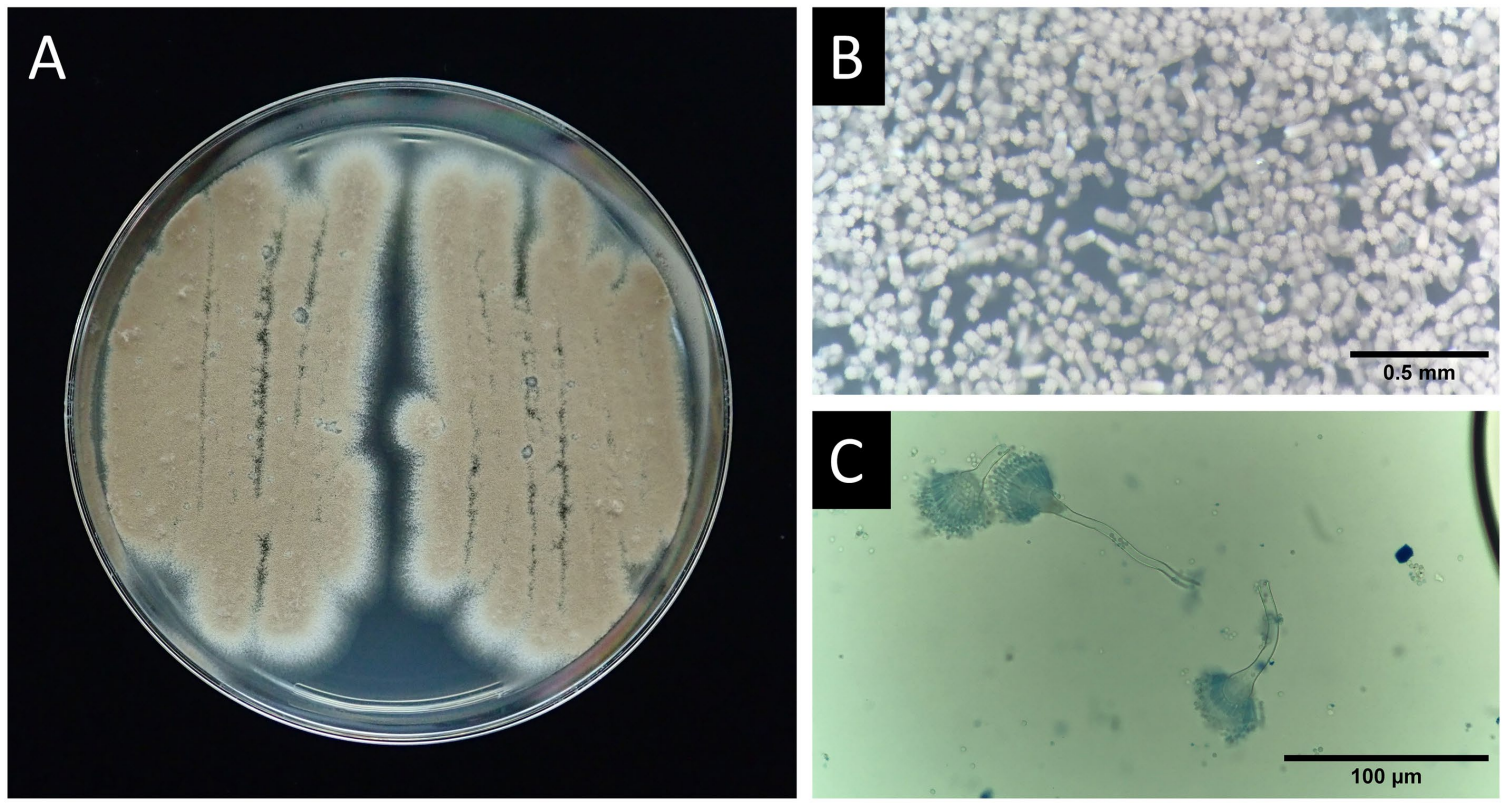
Morphological presen tation of *A. terreus* from case 1. **(A)** Colony morphology on PDA plate (petri dish diameter: 90 mm). **(B)** A stereomicroscopic image showing dense clusters of conidial heads stained with lactophenol cotton blue solution. Scale bar represents 0.5 mm. **(C)** Conidial heads of the isolate. Scale bar indicates 100 μm.

From the samples of the Case-1 Okinawa Rail, several cinnamon-brown colonies were grown on PDA (Figure 3A). After culturing for 4 days, column-like conidial heads were observed under a stereomicroscope (Figure 3B). The conidial heads exhibited a typical aspergillum structure (Figure 3C). Although we considered the isolate most likely to be *A. terreus* based on these observations, we further confirmed this using DNA sequencing. The sequences of the internal transcribed spacer (ITS) and the *benA* gene were identical and almost identical (two mismatches were found) to those of the *A. terreus* lectotype strain, respectively. The strain was concluded to be *A. terreus* and was named TT0037.

**Figure 4:**
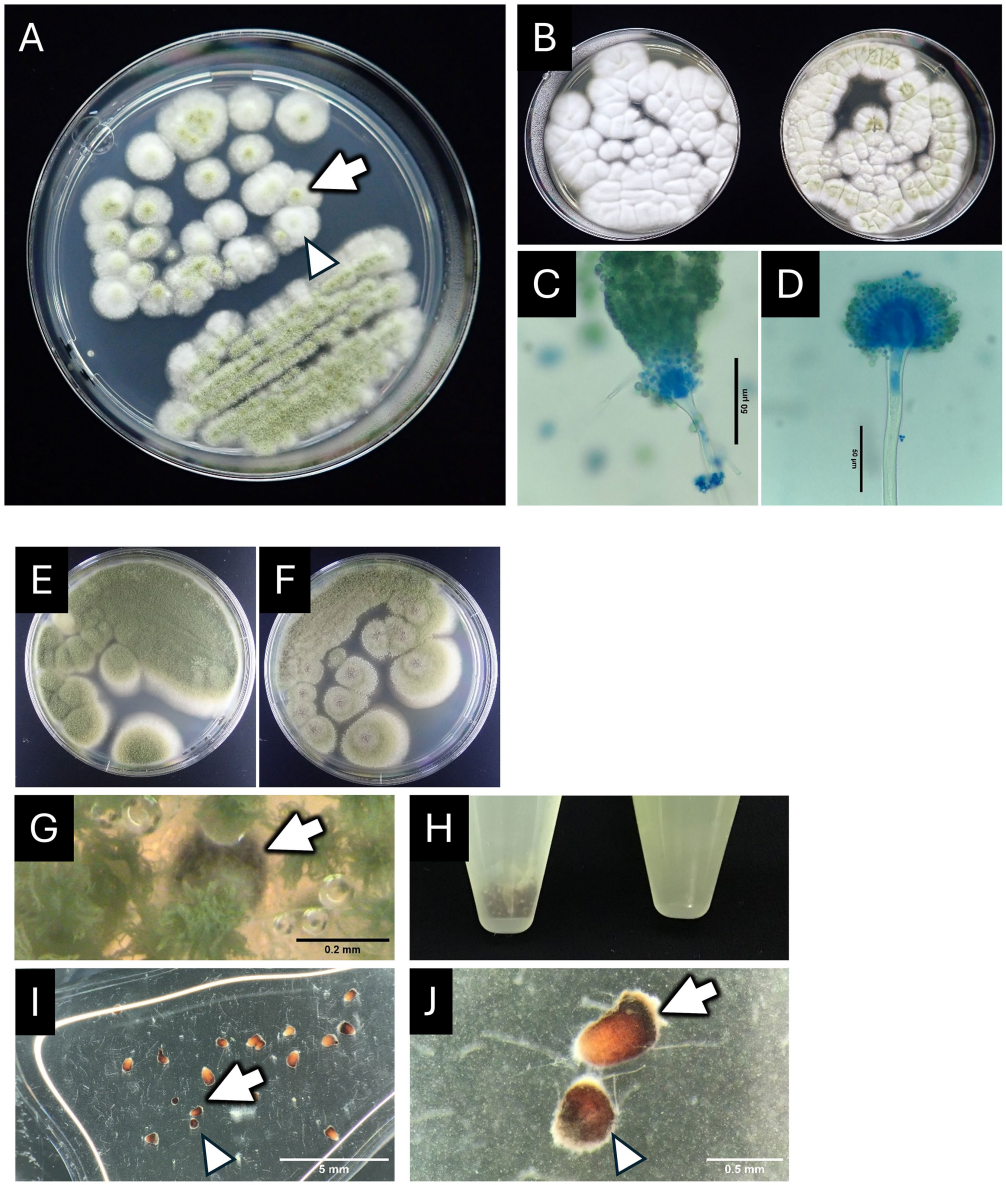
Morphological presentation of the *A. pseudonomiae* isolates from a fungal mass of case 2. **(A)** Colony morphology on a PDA plate shows diverse coloration from white to green. Arrow and arrowhead indicate a representative colony with a strong green center (the S-type isolate) and a representative colony with a weaker green center (the NS-type isolate), respectively. **(B)** Colony morphology on SDA plates: the left plate shows colonies of the NS-type strain, and the right plate shows colonies of the S-type strain. **(C,D)** Conidial heads of the NS-type isolate **(C)** and the S-type isolate **(D)** were stained with lactophenol cotton blue solution. The scale bar represents 10 μm. **(E,F)** Colony morphology on PDA plates: the plate in panel **E** shows colonies of the NS-type strain, and the plate in panel **F** shows colonies of the S-type strain. **(G)** Brown-black sclerotium-like particle (indicated by arrow). **(H)** Spore suspensions from S-type strain (the left tube) and NS-type strain (the right tube) in 15-mL conical tubes. Brown-black sclerotium-like particles were found as sediment in the left tube. **(I,J)** Stereomicroscopic observation of the sclerotium-like particles. Panel **(J)** Is an enlarged image of two particles designated with an arrow and an arrowhead. These petri-dishes (panels **A,B,E,F**) were 90-mm diameter.

From the Case-2 Okinawa Rail samples, two types of colonies were identified on PDA by macroscopic observation (Figure 4A). One type showed good conidiation with a bright green color on the colonies (Figure 4A, arrow). The other type exhibited reduced conidiation (Figure 4A, arrowhead). The phenotypic difference was clearer on Sabouraud dextrose agar (SDA, Figure 4B). Both isolates exhibited typical aspergillum structures, as shown in Figures 4C,D. In addition to the difference in conidiation (Figures 4E,F), the isolate with good conidiation showed a sclerotium-like structure on PDA (Figures 4F–J). The ITS sequences of the two isolates were identical. Moreover, their ITS sequences were highly similar to those from the type strains of *A. nomiae* NRRL 13137 (one mismatch) and *A. pseudonomiae* NRRL 3353 (two mismatches). For further identification, the partial sequence of *benA* was analyzed. The *benA* sequences of both isolates were also identical, and they were matched completely to the sequence of *A. pseudonomiae* NRRL 3353 but not that of *A. nomiae* NRRL 13137 (10 mismatches in the 445 nucleotides). Based on these data, these isolates were identified as *A. pseudonomiae*, and the strain forming spores and sclerotia was designated as the sclerotium-forming (S)-type, while the strain forming less spores was designated as the non-sclerotium-forming (NS)-type strain.

### Low azole susceptibility of *Aspergillus terreus*

The antifungal susceptibilities of these isolates were examined, and the results are presented in Table 1. Minimum inhibitory concentration (MIC) values of amphotericin B and itraconazole against the *A. terreus* isolate obtained in Case 1 were comparable to their geometric mean MICs against the six *A. terreus* strains reported in our previous study (27). The MIC of voriconazole against the *A. terreus* isolate was 2 μg/mL. The value was higher than those for the six *A. terreus* isolated in our previous study (27), for which the MICs of voriconazole were 0.25 and 0.5 μg/mL.

**Table 1:** Antifungal susceptibility of isolates from Okinawa Rails.

	MEC (μg/mL)		MIC (μg/mL)		
Strain	MCFG	CPFG	AMPH-B	ITCZ	VRCZ
*A. terreus* TT0037	≤0.015	0.125	1	0.25	2
*A. pseudonomiae* NS-type isolate	≤0.015	0.125	1	0.25	0.5
*A. pseudonomiae* S-type isolate	≤0.015	0.125	2	0.25	0.5

MEC, minimum effective concentration; MIC, minimum inhibitory concentration; MCFG, micafungin; CPFG, caspofungin; AMPH-B, amphotericin B; ITCZ, itraconazole; VRCZ, voriconazole.

The genome assembly showed 99.4% completeness. Statistical values by Benchmarking Universal Single-Copy Orthologs (BUSCO) analysis are shown in Table 2. Comparisons of Cyp51A, Cyp51B, Cdr4, Cdr4B, AtrR, FfmA, Hmg1, and HapE of *A. terreus* TT0037 revealed an amino acid substitution (Asn24His) in AtrR of the TT0037 strain and 11 differences in the amino acid sequences of Cdr4 at Ser7Phe, Trp8Gly, Pro159Arg, Ala174Thr, Arg242Gln, Ile270Val, Val273Gly, Asn568Asp., Thr621Ala, Asp922Tyr, Glu1127Lys, and Val1292Ala. Furthermore, Cdr4 of strain TT0037 had a stop codon at position 56 (Glu56Stop). In Cdr1B of *A. terreus* TT0037, Met1278Ile polymorphism was found compared to the reference sequence.

**Table 2:** Summary of genome analysis.

Species strain	*A. terreus* TT0037	*A. pseudonomiae* NS-type APSETT444	*A. pseudonomiae* S-type APSETT445
Read N_50_ (bp)	10,155	11,084	11,323
Contig	12	11	14
Contig N_50_ (bp)	4,258,709	4,325,814	4,299,806
The longest contig	5,035,756	6,098,594	4,939,587
Total (bp)	29,917,180	36,877,886	36,881,579
GC contents (%)	52.3	47.9	47.9
Coverage	41.7	33.7	33.7
BUSCO			
Complete and single-copy	4,151	4,145	4,144
Complete and duplicated	15	11	11
Fragmented	2	0	1
Missing	23	35	35

### Aflatoxigenicity of *Aspergillus pseudonomiae*

**Figure 5:**
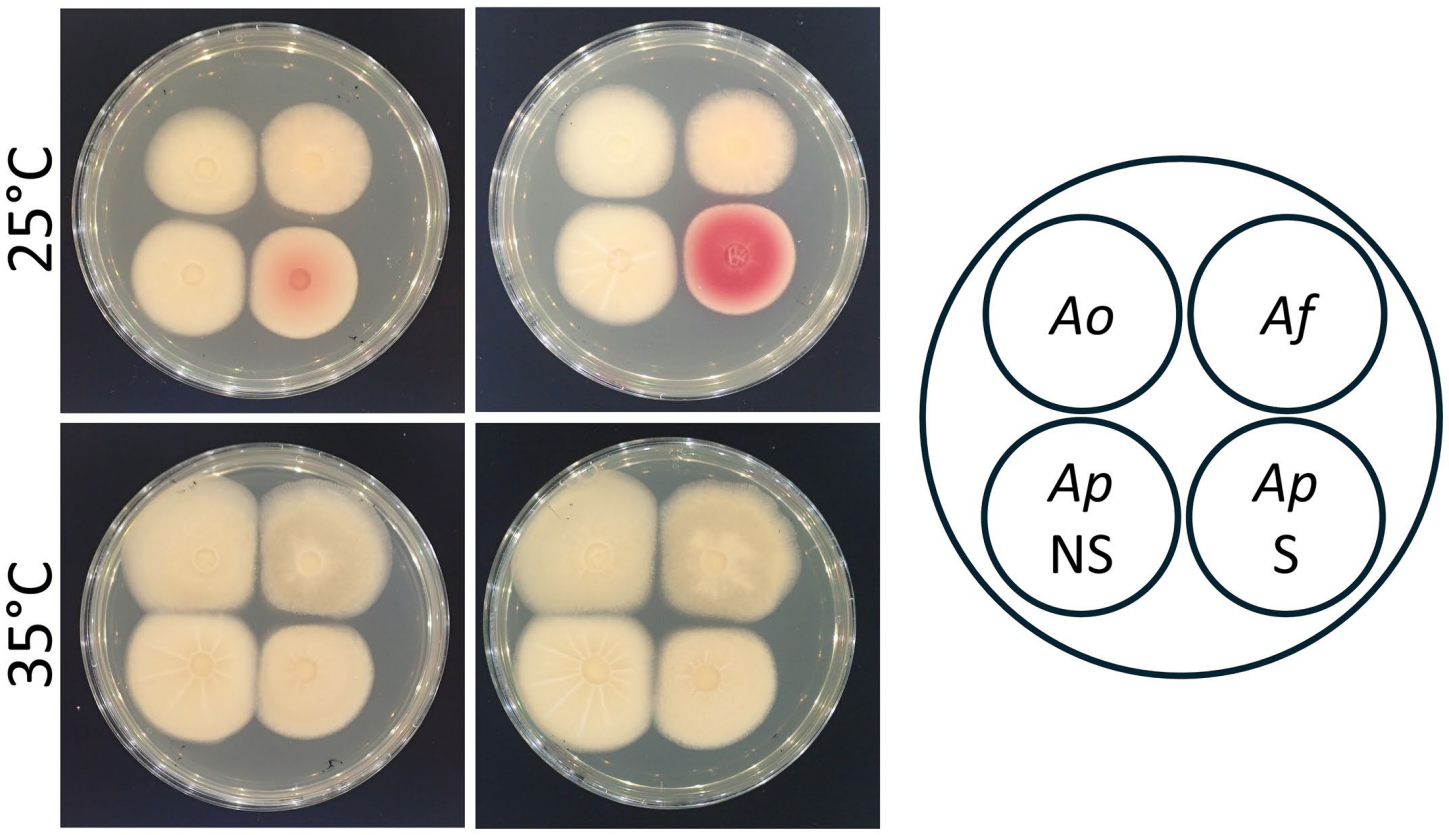
Aflatoxigenicity of NS- and S-type isolates of *A. pseudonomiae*. The layout of four fungal colonies is shown on the right of this figure. Ao: *Aspergillus oryzae* RIB40, a non-aflatoxin producer; Af: *Aspergillus flavus* NRRL 3357, an aflatoxin producer; Ap NS: *Aspergillus pseudonomiae* NS-type strain; Ap S: *Aspergillus pseudonomiae* S-type strain. Dichlorvos was added to the plates in the right column to visualize aflatoxigenicity. Plates incubated at 25 °C and 35 °C for 3 days are shown in the top and the bottom rows, respectively.

The S-type strain of *A. pseudonomiae* showed intense red colonies by the dichlorvos-ammonia (DV-AM) method after culturing at 35 °C but not at 25 °C, indicating that the S-type strain was an aflatoxigenic fungus (Figure 5). In contrast, aflatoxigenicity was not observed in the NS-type strain. Additionally, the *A. terreus* strain isolated from Case 1 did not show aflatoxigenicity. Furthermore, we quantified aflatoxin production in culture filtrates from the NS-type- and S-type strains. Upon culturing at 25 °C, aflatoxins were not detected in the culture filtrate from the NS-type strain; however, 51 ng/mL of aflatoxins were detected in the culture filtrate from the S-type strain. Aflatoxin production at 35 °C was not detected in the NS-type and S-type strains. Upon further analysis with LC–MS, aflatoxin B_1_ (0.7 ng/mL) and aflatoxin G_1_ (0.6 ng/mL) were detected in the culture filtrate of the S-type strain but not the NS-type strain, cultured at 25 °C. In Case 2, the S-type- and NS-type strains were isolated from the same host, but these strains showed different phenotypes with respect to aflatoxin production.

**Figure 6:**
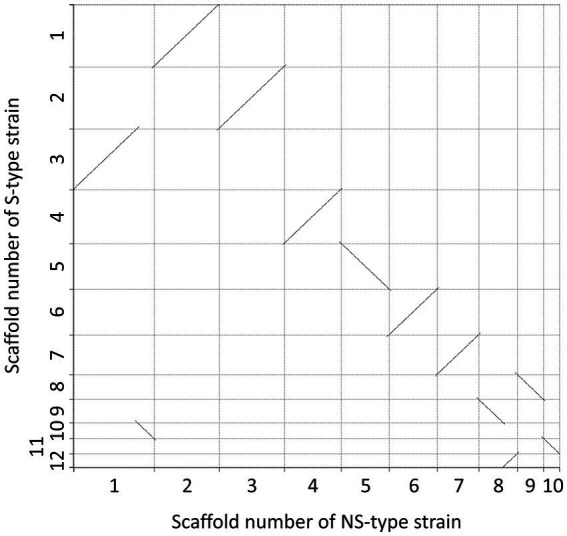
Scaffold comparison between NS-type strain and S-type strain. This scatter plot illustrates the scaffold comparison between the NS-type strain (x-axis) and the S-type strain (y-axis). Each dot represents a scaffold shared between the two strains, with its position corresponding to the scaffold’s length or coordinate in each genome. The diagonal lines indicate regions of synteny or conserved genomic structure.

**Figure 7:**
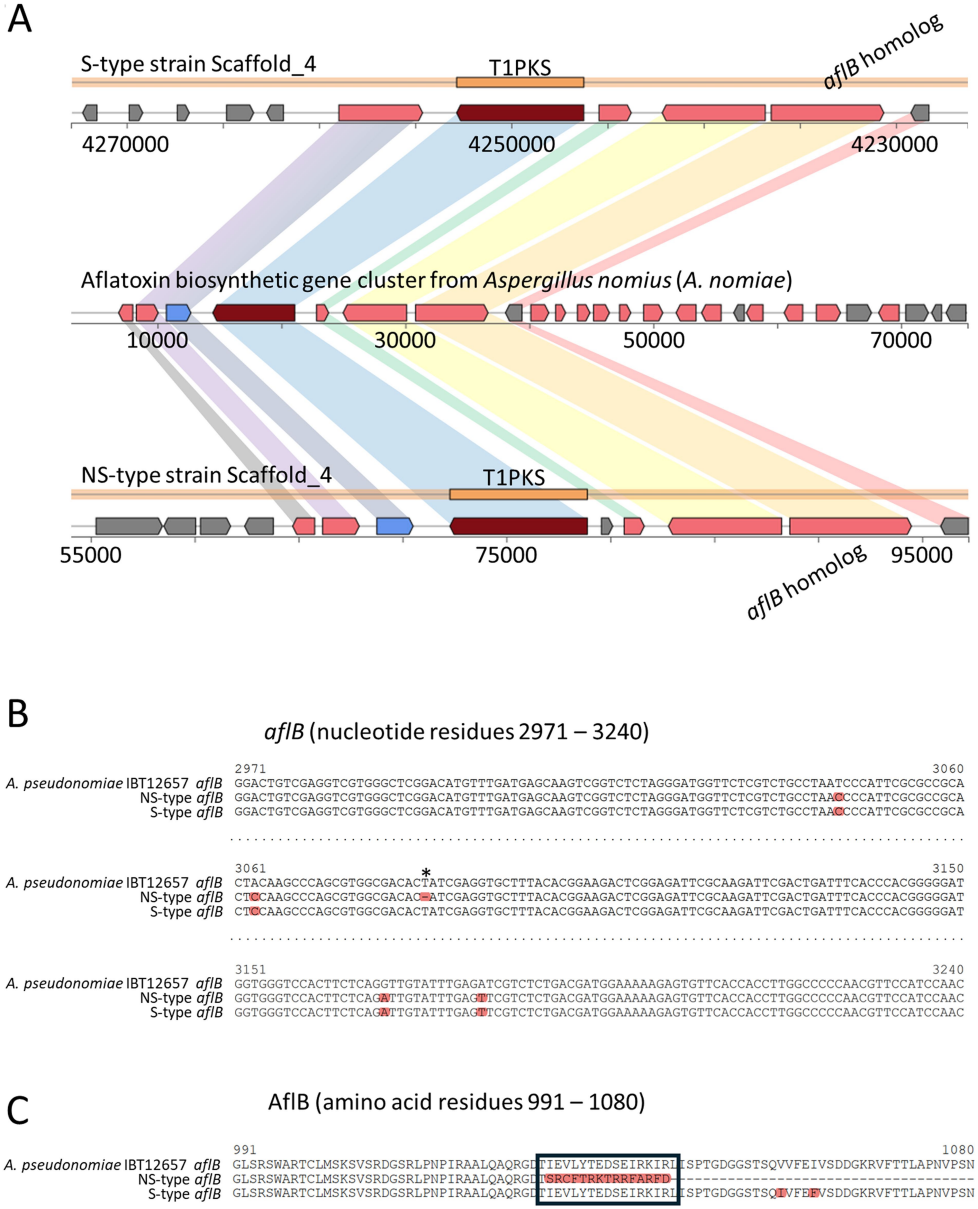
Comparison of aflatoxin biosynthetic gene clusters from *Aspergillus nomiae* (BGC0000009) and *A. pseudonomiae* S-type and NS-type strains. **(A)** Comparison among putative aflatoxin biosynthetic gene clusters from *Aspergillus nomiae* (BGC0000009) and *A. pseudonomiae* S-type (Scaffold_4) and NS-type strains (Scaffold_4). **(B,C)** Deletion sites in *aflB* gene **(B)** and AflB protein **(C)** of *A. pseudonomiae* NS-type, where single nucleotide deletions were found, are indicated by asterisks **(B)**. Truncated sequence is indicated by a black frame **(C)**.

The assembled genomes of both strains presented 99.2% completeness, and a summary of the genome analysis of *A. pseudonomiae* NS-type- and S-type strains is shown in Table 2. A comparison of the contigs obtained from each strain showed no large inversions, deletions, or insertions (Figure 6; Supplementary Figures S1, S2). A comparison between these mitochondrial genomes revealed only one nucleotide deletion in the NS-type strain (Supplementary Figure S2). The deleted nucleotide was retained in the mitochondrial genome of a previously reported *A. pseudonomiae* CBS 119388 strain (accession number: NC_066212). Secondary metabolite biosynthetic gene clusters were predicted using antiSMASH, and clusters similar to an aflatoxin biosynthetic gene cluster from *Aspergillus nomius* (MIBiG accession BGC0000009) were found in the S-type- and NS-type strains (Figure 7A). These data suggest that the S-type- and NS-type strains have almost identical genomes.

A nucleotide deletion in the region encoding AflB, also known as Fas-1 and HexB, in the NS-type strain, was found in the 44-kb putative aflatoxin biosynthetic gene cluster (Figure 7B). This deletion led to the production of truncated AflB (Figure 7C), suggesting that the impairment of AflB results in an inability to produce aflatoxin in the NS-type strain.

## Discussion

This study describes two cases of aspergillosis in Okinawa Rails. *Aspergillus* is a major infectious threat to birds, particularly domestic and wild flightless species. Environmental conditions, along with intrinsic factors associated with respiratory anatomy, physiology, and immune function, constitute primary risk factors for the infection of avian species by this opportunistic fungus (10, 19). Pathological examination revealed adenocarcinoma in Case 1, as described in the Supplementary Text. The tumor was considered to have led to immunosuppression and nutritional decline, resulting in the deterioration of the overall condition and rapid progression of aspergillosis.

Contact with soil was reported to be related to the occurrence of aspergillosis cases in penguins (19). Similarly, Okinawa Rails may also be vulnerable to contracting the disease due to likely exposure to native *Aspergillus* spp. in soil; however, no cases of aspergillosis have yet been documented in free-living animals to date. The ubiquitous *Aspergillus fumigatus* is the predominant causative agent of aspergillosis. Nonetheless, a case of aspergillosis in captive Okinawa Rail caused by *A. flavus* was reported by Kano et al. (21). *A. terreus* from soil sources has also been identified as an etiological agent in other cases (36–38). Minor or less common causative species of aspergillosis including *A. terreus* have recently increased the importance due to differences in their antifungal susceptibility patterns from *A. fumigatus* (39, 40). Climate change-driven shifts in the distribution and abundance of *Aspergillus* species raise concerns about potential impacts on avian and human health, as altered fungal prevalence in soil and habitats may increase exposure risk for birds, including endangered species (41). Heat stress associated with global warming may further compromise the health of wildlife, including endangered species, potentially increasing their susceptibility to infections caused by *Aspergillus* species.

Azole resistance has been principally reported in *A. fumigatus* isolates worldwide. A recent retrospective study on resistance surveillance used a collection of clinical *A. fumigatus* isolates from various animal species collected over five years in the Netherlands (42). It found that 15% (2/13) of birds presented *A. fumigatus* azole-resistant isolates harboring *cyp51A*-mediated mutations (42). On the other hand, Silvanose reported that *A. terreus* was isolated from 13 falcons (29%) diagnosed with fungal diseases of the lower respiratory tract (40, 41), indicating that this species is also common among animals. The report further showed that all *A. terreus* isolates were susceptible to voriconazole (with an MIC of less than 1 μg/mL) before and during voriconazole treatment. Dietl also indicated that all environmental isolates were susceptible to voriconazole; however, 3.9% of clinical isolates were resistant (with an MIC of 2 μg/mL or more) (42, 43). *A. terreus* isolate from Case 1 showed high MIC (2 μg/mL), but whether the increase was native or occurred during the treatment remains unknown. Although *cyp51A* mutation and tandem repeat acquisition in the promoter are the most frequent mechanisms for acquiring azole resistance, such alterations were not observed in the isolate.

Sequence alignment of the AtrR protein in *A. terreus* TT0037 revealed a non-synonymous polymorphism at residue 24 (Asn24His). AtrR is a Zn_2_-Cys_6_ transcription factor and an essential determinant of azole resistance in *Aspergillus fumigatus* (43, 44). In recent years, reports have highlighted the role of *AtrR* in *A. fumigatus* as a determining factor in the development of azole resistance by co-regulating the expression of *cyp51A* and non-*cyp51A* elements, such as *cdr1b*, which might trigger and compromise susceptibility to azole drugs (43, 44). This substitution (Asn24His) was previously reported in an azole-resistant *A. terreus* strain (44–46). Although AtrR contains two conserved domains (GAL4 and fungal_TF_MHR), the 24th residue lies outside these regions. Its potential role in azole susceptibility remains unclear and warrants further investigation. Studies on *Aspergillus* species have demonstrated that alterations in the genome, such as the loss and overexpression of ABC transporters, cause hypersensitivity and elevated resistance to azoles, respectively (45–47). Cdr1B is a well-known efflux transporter implicated in azole resistance. A substitution (Met1278Ile) was identified between the Cdr1B sequence of reference *A. terreus* strain and that of the strain TT0037. However, two out of three publicly available *A. terreus* Cdr1B sequences (accession numbers are XP_001210721.1, GES59343.1, and KAG2415106.1) also contain Ile at this position, suggesting that this variation is likely unrelated to azole resistance. Cdr4 had a stop codon at the position of the 56th residue and many substitutions were located in the coding sequence. The stop codon might have rendered the protein nonfunctional, allowing subsequent accumulation of mutations in the gene. Cdr4 (ATEG_05640) and the homologs including Cdr4B (ATEG_07138) have been described as candidate proteins that contribute to azole resistance in *A. terreus* (48). AbcB (Accession number XP_752803.1), the second closest homolog of *A. terreus* Cdr4 in *A. fumigatus*, has been shown to play a role in azole resistance (46). Interestingly, the *abcB*-deleted strain exhibited increased susceptibility to azoles (46), whereas our azole-tolerant isolate showed a largely truncated Cdr4, which appears contradictory. Further studies are needed to clarify the impact of Cdr4 impairment on antifungal susceptibility. The mechanisms of azole resistance in strains with non-*cyp51A* mutations remain poorly understood. To elucidate the resistance mechanism of the isolate, further comparisons of genomic alterations using additional *A. terreus* strain genomes are required.

*A. pseudonomiae* (earlier known as *A. pseudonimius*) belongs to the *Aspergillus* section *Flavi* (49). The section *Flavi* includes *A. flavus* and *A. nomiae* (previously known as *A. nomius*), which are well-known aflatoxin producers (50, 51). *A. flavus* is the most prevalent causative agent of aspergillosis in tropical countries (52, 53). *A. pseudonomiae* has been detected around the world (49, 54–57). Recently, a report by Tachikawa showed the presence of *A. pseudonomiae* in the soil environment of the Ogasawara Islands, Japan (58).

Although this species has been isolated from human patients (59–61), to the best of our knowledge, no reports of its isolation from animals exist. Importantly, our data suggest that *A. pseudonomiae* is native to Okinawa Island in Japan and is one of the most frequent causative agents of aspergillosis among avian species, including the Okinawa Rail. Isolates from Case 2 showed two types of colonies, one of which, the S-type strain, formed sclerotia and was aflatoxigenic. The reason for the coexistence of two strain types within a single individual is not fully understood. Possible explanations include separate infections by different phenotypes or phenotypic divergence during infection. Genomic analysis in this study showed a limited number of nucleotide polymorphisms between the two *A. pseudonomiae* strains. These data suggest that the analyzed strains were derived from the same ancestor. The NS-type strain did not show sclerotia formation. Sclerotia are hardened, melanized structures that function as survival structures. Sclerotia formation is presumed unnecessary when an infection develops within a host. In addition, aflatoxin production is impaired in the NS-type strain. The aflatoxin production in the S-type strain was repressed at 35 °C, suggesting that aflatoxin is not produced from *A. pseudonomiae* during the infection in Okinawa Rail. At some point during infection, an NS-type strain that lost its aflatoxin-producing capacity may have been derived.

The *A. pseudonomiae* NS-type strain has nucleotide deletion in *aflB* gene. The AflB is a fatty acid synthase beta subunit located within the aflatoxin biosynthetic gene cluster, which is involved in the synthesis of the polyketide backbone from the primary metabolite (62, 63). StcK, a homolog of AflB in *A. nidulans*, is required for the synthesis of sterigmatocystin, a mycotoxin related to aflatoxins (64). A single-nucleotide deletion led to the production of truncated AflB, resulting in the loss of AflB function and aflatoxin production capacity.

In summary, this study reports on aspergillosis in the Okinawa Rail, an endangered flightless bird restricted to the northern forest areas of Okinawa Island. Two cases of aspergillosis were identified in captive Okinawa Rails, with *Aspergillus terreus* and *Aspergillus pseudonomiae* identified as the causative fungi. One bird died of an infection caused by *A. terreus*, which showed low susceptibility to voriconazole; however, the mechanism of low azole susceptibility remains unclear. Another bird died of an infection caused by *A. pseudonomiae*. The isolates exhibited two phenotypes based on the presence or absence of sclerotia formation and aflatoxin production. Genome analysis suggested that both phenotypes were derived from the same ancestral strain. This is the first study to report the detection of *A. pseudonomiae* in animal hosts in Japan. Identifying the causal agents of aspergillosis in Okinawa’s northern forests is essential for understanding disease ecology, and assessing potential threats, and developing appropriate countermeasures to protect the Okinawa Rail.

## Data Availability

The obtained sequence data were deposited in DNA Data Bank of Japan/European Nucleotide Archive/Genetic Sequence Database (DDBJ/ENA/GenBank) under accession numbers DRR582540, DRR582541, and DRR582542 for *Aspergillus terreus* TT0037 strain, *Aspergillus pseudonomiae* NS-type strain (APSETT444), and *Aspergillus pseudonomiae* S-type strain (APSETT445), respectively. The assembled scaffolds with annotations were deposited under accession numbers BAAFRR010000001-BAAFRR010000012, BAAFRP010000001-BAAFRP010000011, and BAAFRQ010000001-BAAFRQ010000014, respectively.
