# Supplementary material for: Identification of Aspergillus terreus and Aspergillus pseudonomiae as causative agents of aspergillosis in endangered Okinawa Rails
Source: Front Vet Sci. 2025 Dec 22;12:1675145. doi: 10.3389/fvets.2025.1675145 (PMC12766970; doi:10.3389/fvets.2025.1675145)
Supplement: Supplementary file 1 [file Table_1.DOCX]

| Table S1. The monitored ions and their respective transitions with CE to detect aflatoxins. | |
| --- | --- |
| Target compound | *m*/*z* (CE) |
| Aflatoxin G_1_ | 329.0 > 243.0 (CE: －27 V) |
| Aflatoxin G_2_ | 331.0 > 245.0 (CE: －31 V) |
| Aflatoxin B_1_ | 313.0 > 241.0 (CE: －37 V) |
| Aflatoxin B_2_ | 315.0 > 259.0 (CE: －28 V) |
| Aflatoxin M_1_ | 329.0 > 273.0 (CE: －25 V) |
| CE, collision energy |  |
