# Supplementary material for: Identification of Aspergillus terreus and Aspergillus pseudonomiae as causative agents of aspergillosis in endangered Okinawa Rails
Source: Front Vet Sci. 2025 Dec 22;12:1675145. doi: 10.3389/fvets.2025.1675145 (PMC12766970; doi:10.3389/fvets.2025.1675145)
Supplement: Supplementary file 2 [file Table_2.DOCX]

## **Two aspergillosis cases in Okinawa Rails: History and treatment details**

**Case 1:**

Case 1 involved a 13-year-old male Okinawa Rail (Pedigree Registration Number: SB34). Since September 2019, occasional moist breath sounds were heard, prompting chest X-ray and blood tests, which revealed pneumonia in the right lung and mild cardiomegaly. Treatment with antibacterials cured the respiratory symptoms and pneumonia. From January 1, 2020, weight loss followed by a decrease in appetite was observed. On January 25, examination revealed respiratory distress during restraint and noise in the right chest, leading to the initiation of treatment with enrofloxacin (8 mg/kg SID per os) and itraconazole (10 mg/kg SID per os) from January 25 and January 26, respectively. On February 4, because an X-ray examination revealed air sac lines, itraconazole was replaced with voriconazole (40 mg/kg BID per os from February 5 to the morning of March 6 and then 40 mg/kg BID subcutaneously from the afternoon of March 6 to the morning of March 11). Micafungin sodium (10 mg/kg via a subcutaneous route) was administered from March 6 to 29 (BID) and from March 30 to April 14 (SID). Amphotericin B (50 mg) was administered by nebulization (SID) from the afternoon of March 11 to March 12 and then BID from March 13 to the morning of March 27. Subsequently, the weight gradually decreased, and the respiratory condition worsened, necessitating hospitalization and treatment in the ICU with high-concentration oxygen. On April 14, due to a lack of improvement in respiration and progression of cardiomegaly with left atrial enlargement observed on X-ray images, treatment with cardiotonics and diuretics was initiated, and antifungal medication was temporarily discontinued. Oxygen inhalation was discontinued on May 15. However, on May 27, weight loss and appetite loss were observed again, leading to the resumption of high-concentration oxygen treatment. From May 31, the respiratory condition worsened, with prolonged sitting periods, and from June 1, the rail began to have difficulty standing. From June 4, the rail was unable to lift his head because of gasping breaths, and it died early in the morning of June 7. Pathological examination revealed adenocarcinoma and putative fungal colonies outside the lung (front and top of the heart, upper part of the right kidney, and the testis), as well as in the air sacs and lungs (Fig 1). A specimen of the fungal mass (Fig 1A, white circle) showed an aspergillum-like structure (Fig 1E). Pathological examination revealed adenocarcinoma of the lung in addition to fungal colonization.

**Case 2:**

Case 2 involved a captive female Okinawa Rail (Pedigree Registration Number: SB146), which died at approximately 2 years and 10 months of age. On January 7, 2021, the rail exhibited labored breathing at rest. On examination, open-mouth breathing and significant respiratory noise were observed. On the same day, X-ray examination revealed a massive object extending from the upper part of the heart to the pericardial area and thickening of the anterior and posterior air sacs. Blood tests revealed dehydration, high levels of bile acids and creatinine kinase, and mild elevation of uric acid levels. Aspergillosis was strongly suspected from the symptoms; therefore, therapy using several antifungals was immediately started in the ICU under high O_2_ condition. Itraconazole (10 mg/kg BID per os from day 1 to day 34) and micafungin sodium (10 mg/kg BID through subcutaneous route on day 1 and 20 mg/kg BID from day 2 to day 5), voriconazole (40 mg/kg BID via intramuscular route from day 6 to day 11), or amphotericin B (2 mg/kg BID via intratracheal route from day 12 to day 15, or 25 to 35 mg SID from day 15 to day 26 and 25 mg BID from day 27 to day 34 with nebulization) were administered for the treatment of aspergillosis. Orbifloxacin (20 mg/kg SID from day 1 to day 5 and 10 mg/kg SID from day 12 to day 34) and cefozopran hydrochloride (20 mg/kg BID from day 6 to day 11) were used as antibacterial agents. On day 31, vomiting was observed, and from day 33, the rail began to sit down after treatment and died on day 34. Necropsy revealed that the thoracic and abdominal cavities, air sacs, and lungs were occupied by large fungal masses (Fig 2). Powdery surfaces with ridges were observed in areas that were assumed to be in contact with air (Fig 2C). Fungal components, including several spores and aspergillum-like structures, were observed in a specimen from the surface (Fig 2D).
