# Supplementary figures and images for: Identification of Aspergillus terreus and Aspergillus pseudonomiae as causative agents of aspergillosis in endangered Okinawa Rails
Source: Front Vet Sci. 2025 Dec 22;12:1675145. doi: 10.3389/fvets.2025.1675145 (PMC12766970; doi:10.3389/fvets.2025.1675145)

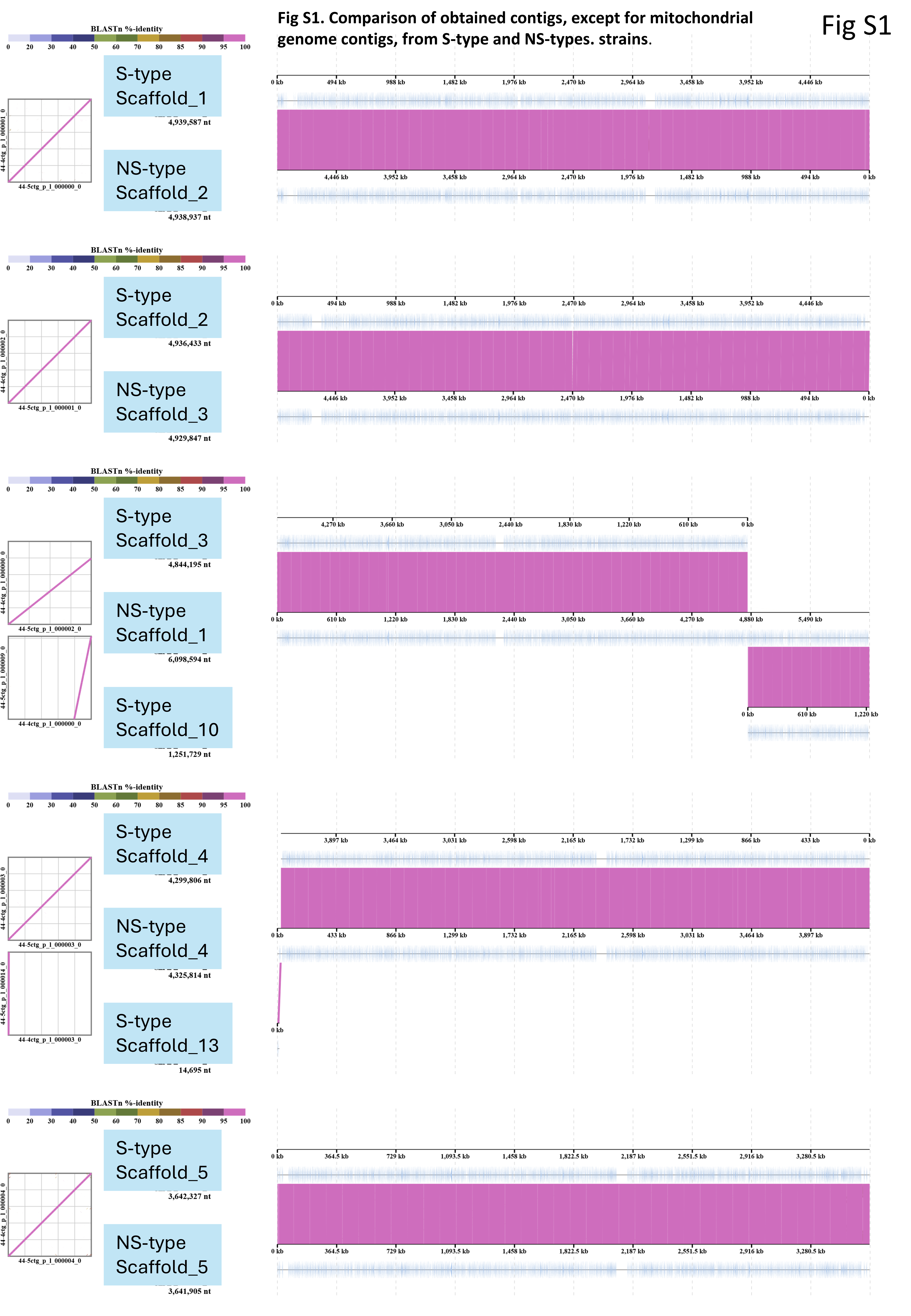

Supplement: Supplementary file 3 [file Image_1.tif]

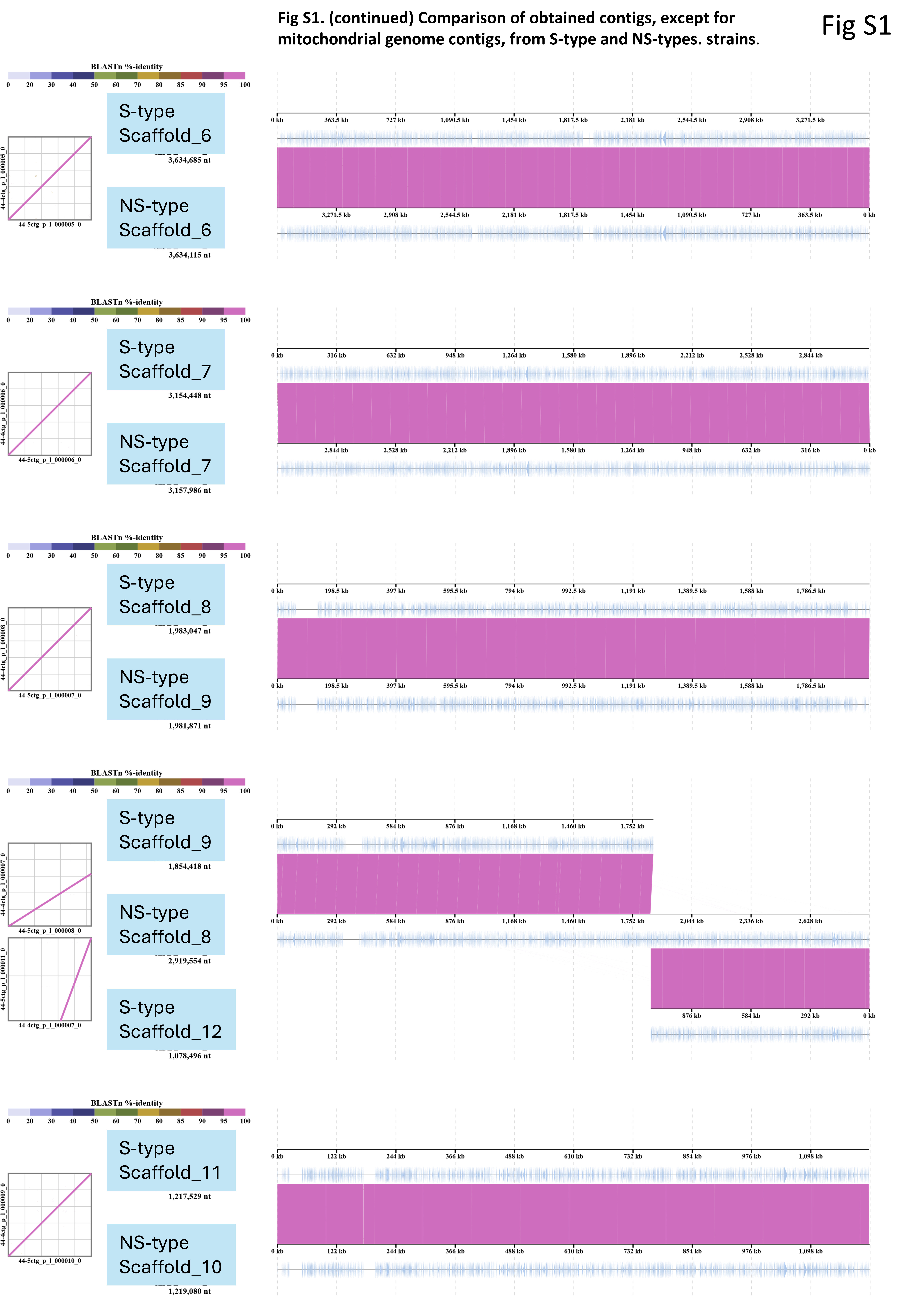

Supplement: Supplementary file 4 [file Image_2.tif]

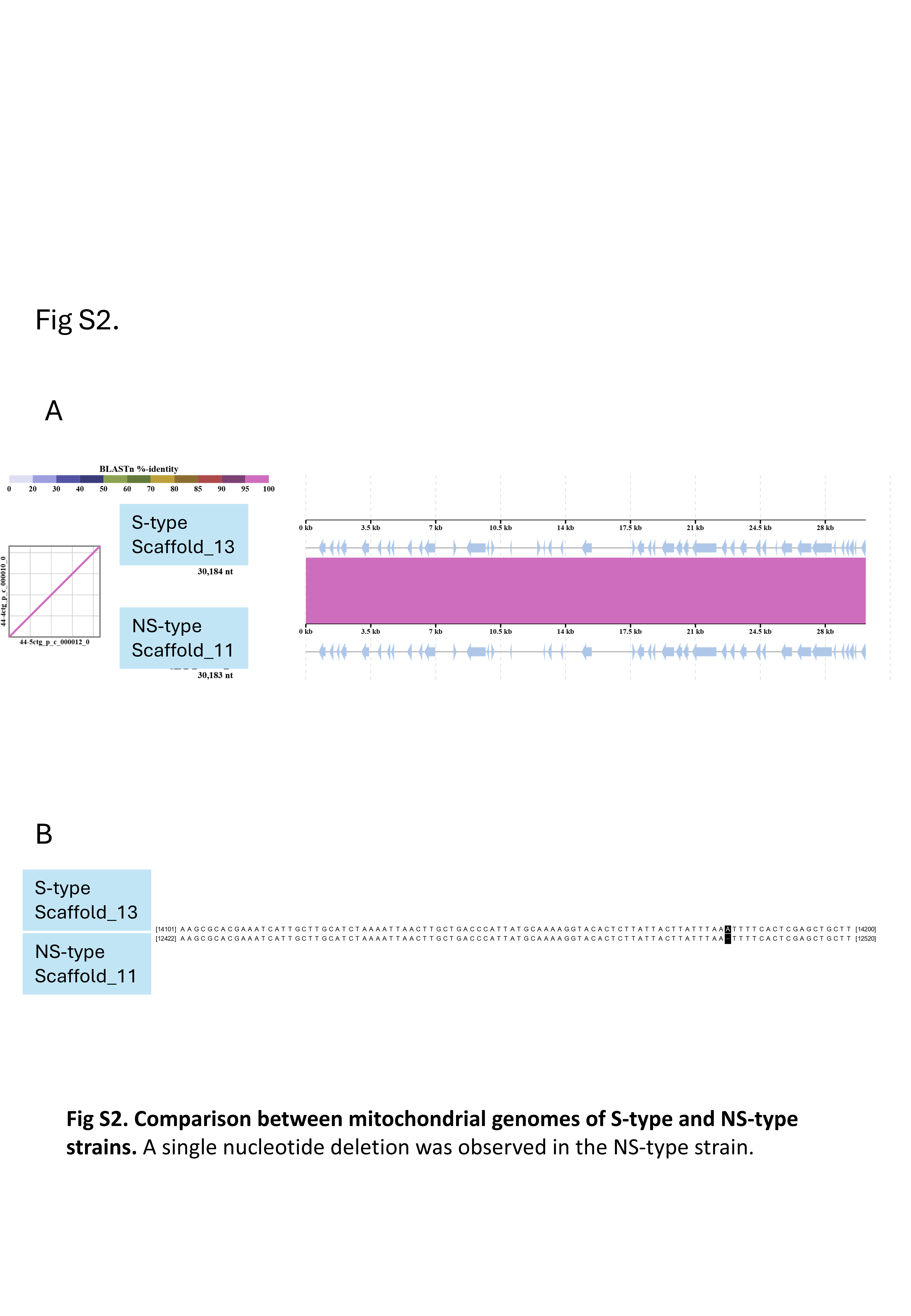

Supplement: Supplementary file 5 [file Image_3.tif]
